# A Comparison of Transvaginal Removal and Repair of Uterine Defect for Type II Cesarean Scar Pregnancy and Uterine Artery Embolization Combined With Curettage

**DOI:** 10.3389/fmed.2021.654956

**Published:** 2021-06-22

**Authors:** Shanshan Cao, Guijing Qiu, Peipei Zhang, Xinyan Wang, Qing Wu

**Affiliations:** ^1^Tiantai People's Hospital of Zhejiang Province, Taizhou, China; ^2^Department of Gynecology, Zhejiang Provincial People's Hospital, People's Hospital of Hangzhou Medical College, Hangzhou, China

**Keywords:** cesarean scar pregnancy, uterine artery embolization, SAC, transvaginal sugery, methotrexate

## Abstract

**Background:** There is no consensus on a standardized therapy for type II cesarean scar pregnancy (CSP II). The objective of the present study was to evaluate the efficacy and safety and compare costs associated with transvaginal removal and repair (TRR) of uterine defect for CSP II to those of uterine artery embolization (UAE) and curettage.

**Methods:** We conducted a retrospective study that included 87 patients diagnosed with CSP II and treated by performing UAE in combination with curettage and hysteroscopy (*n* = 53), or TRR (*n* = 34). Clinical data and outcomes were analyzed.

**Results:** UAE and TRR groups exhibited similar success rates. The TRR group had significantly lower complication rates (30.19 vs. 8.82%, *P* < 0.05) and lower total costs (13,765.89 ± 2,029.12 vs. 9,063.82 ± 954.67, *P* < 0.05) than the UAE group. The anterior myometrium of the lower uterine segment was relatively thicker after performing TRR, and no patient suffered from recurrent CSP II. The proportion of patients in the TRR group who had full-term delivery without uterine rupture was 88.24% (30/34), while four patients failed to pregnancy.

**Conclusion:** TRR is a safe and effective treatment method for patients with CSP II and presents a highly cost-effective outcome, especially for patients with future fertility desire.

## Introduction

Cesarean scar pregnancy (CSP) is a rare type of ectopic pregnancy characterized by implantation of the gestational sac (GS) in the anterior uterine wall of the cesarean scar between the GS and the bladder (1). CSP occurs in one in every 500 pregnancies among women who previously underwent cesarean delivery (2). There are two types of CSP: (i) CSP I, which refers to the implantation of the GS on a previous cesarean scar with progression into the cervico-isthmus and the uterine cavity and (ii) CSP II that is caused by a deep implantation of the amniotic sac into a previous cesarean scar, which is a defect with infiltrating growth into the uterine myometrium and bulges from the uterine surface with a thin or absent myometrial layer. CSP II may lead to life-threatening complications, such as heavy vaginal bleeding and uterine rupture during the first trimester of pregnancy (3, 4).

However, there is no consensus on astandardized therapy for CSP in clinical practice (5). Blocking blood flow to the GS through uterine artery embolization (UAE) results in death of the embryo and reduces bleeding during curettage, and cesarean scar defect lacks an effective repair strategy. We hypothesized that transvaginal removal and repair (TRR) of uterine defect could have a satisfactory therapeutic outcome in patients with CSP II who have future fertility desire. In the present article, we have reviewed our knowledge of TRR and systematically evaluated its feasibility and clinical value in treating uterine defect associated with CSP II when compared to UAE and curettage. Furthermore, we discussed the indications of different treatments to optimize therapeutic strategies for CSP.

## Materials and Methods

### Patient Selection and Data Collection

The present retrospective study was approved by the ethics committee of the Tiantai People's Hospital of Zhejiang Province. Data were obtained from the hospital's clinical database. A total of 87 patients with CSP II were admitted after cesarean section at the facility from June 2012 to December 2017. Informed consent was obtained from all patients, and they were briefed on the benefits and potential risks of the procedure. Patients with ruptured uteri, heavy vaginal bleeding, abnormal coagulation, severe cardiac, lung, kidney, liver disease, acute inflammation, or other surgical contraindications were excluded. All procedures were performed by the same clinical team, and the procedures were carried out in accordance with relevant guidelines and regulations.

### Diagnosis of CSP II

All patients had a history of previous cesarean section. CSP II was diagnosed based on a positive pregnancy test and transvaginal sonography. Ultrasound diagnostic criteria were as follows (3, 4, 6): (1) an empty uterine cavity and cervical canal without contact with the GS; (2) patients with GS located at the anterior wall of the isthmic segment with or without a cardiac activity; (3) a thin or absent myometrial layer between the bladder and the GS; (4) functional trophoblastic/placental circulation around the GS or mass; and (5) fibrotic tissue implanted in the scar protruding from the uterus and growing toward the abdominal cavity.

### UAE and Curettage

UAE combined with local methotrexate (MTX) infusion, hysteroscopy, and curettage was performed (7). We selected the right transfemoral approach for artery access, and the uterine artery was selectively catheterized with a 5F Yashiro catheter (Terumo, Tokyo, Japan) and embolized with gelatin sponge particles of sizes ranging 1,000–1,400 μm (Alicon Co. Ltd., Hangzhou, China). MTX (25 mg) was bilaterally infused into each uterine artery prior to the embolization procedure. Angiography was conducted to confirm whether occlusion of blood flow was complete. Traditional curettage was initially performed, followed by hysteroscopy after 24–72 h.

### TRR of Uterine Defect for CSP II

TRR was performed under spinal–epidural anesthesia. All patients were placed in a dorsal lithotomy position and their bladders emptied. The cervix was exposed and pulled down as much as possible using two cervical clamps. Adrenaline (10–20 mL) containing 0.3 mg adrenaline/10 mL normal saline was injected submucosally at the level of the cervicovaginal junction for hydrodis section. An incision was made at the anterior cervicovaginal junction, and the anterior drawing hook was inserted into the vaginal incision to retract the bladder upward. The cervix was pulled outward until the lower segment was fully exposed. CSP was identified as a “purplish-blue bulge” located in the anterior part of the lower uterine segment. A transverse incision was made over the prominent site of the bulge. CSP tissue was removed and suction curettage performed on the uterus isthmus through the incision. The edges of the incision were trimmed with scissors, and the myometrial layer was closed with an intermittent suture of 2–0 absorbable sutures and a secondary continuous suture of 2–0 absorbable sutures. The vaginal incision was closed using continuous sutures consisting of 2–0 absorbable sutures. Three strips of gauze were soaked with iodine and paraffin oil (disinfectant) and packed into the vagina to press the incision. Subsequently, the gauze was removed 1 day after the operation. A urinary catheter was maintained postoperatively for 24 h.

### Cost Analyses

Direct and indirect costs associated with TRR and UAE combined with curettage were calculated and analyzed in the present study. The direct costs included the cost of medication, clinical procedure, patient care, nursing ward, clinical materials, and non-medical costs. The indirect costs included loss of work time for the patients and accompanying persons.

### Patient Follow-Up

Successful treatment was defined as complete recovery without severe complications such as heavy vaginal bleeding (loss of more than 200 mL of blood), gastrointestinal perforation, and uterine rupture without second-line therapy (8). We observed the patients, estimated blood loss, and recorded complications (including fever, vomiting, heavy vaginal bleeding, hematoma, retained CSP, infections associated with vaginal incisions, poor healing, bladder trauma, vesicovaginal fistulas, and pain). Telephone reviews or follow-up clinics were recommended. Serum β-hCG levels were monitored weekly until they reached normal levels. The patients used contraceptive methods for 12 months. Ultrasound was used to determine if serum β-hCG levels had normalized. The thickness of the endometrium (TE), thickness of the anterior lower uterine segment myometrium (TM), and uterine adhesion were assessed using ultrasound after 3–6 months.

### Statistical Analysis

Continuous and ordinal data were presented as means ± standard deviation; categorical data were presented as counts and percentages. Demographic, baseline clinical characteristics, and cost analyses were performed using two-sample *t*-tests with normal distribution. The non-parametric test was used when the distribution of data was skewed and not normal. A Chi-square test was used to compare categorical data, and Fisher's exact test was applied when *n* <5. Data were analyzed using SPSS 17.0 software (SPSS Inc., Chicago, IL, USA). All statistical tests were two-tailed, and a *P*-value of < 0.05 was considered statistically significant.

## Results

### Baseline Clinical Characteristics of Patients With CSP

Fifty-three female patients underwent UAE combined with uterine curettage, whereas 34 female patients underwent TRR. All patients had previous cesarean deliveries. Demographic data and baseline clinical characteristics of patients are summarized in Table 1. No statistically significant differences were observed in gestational age, time interval from last cesarean section, serum β-hCG levels, myometrium thickness, and the anterior lower uterine segment myometrium thickness before administration of treatment between the TRR and UAE groups. The TRR group had the desire for future pregnancy, and their ages were lower than those of the UAE group.

**Table 1:** Baseline clinical characteristics of participants before surgery.

**Characteristics**	**UAE group (*n* = 53)**	**TRR group (*n* = 34)**	***P***
Patient age (years)	34.79 ± 3.43	33.08 ± 2.71	0.016
No. of previous cesarean sections	1.60 ± 0.53	1.47 ± 0.56	0.268
Interval time from recent cesarean section (years)	3.64 ± 1.74	2.99 ± 1.29	0.067
Gestational age (d)	49.43 ± 6.38	50.20 ± 6.28	0.581
Serum β-hCG (U/L)	22,322.23 ± 17,296.52	24,077.35 ± 16,590.85	0.640
Diameter of the gestational sac (mm)	28.49 ± 8.12	30.32 ± 7.21	0.287
TE (mm)	10.19 ± 2.40	9.68 ± 2.25	0.323
TM (mm)	1.20 ± 0.59	1.33 ± 0.53	0.280

*TE, thickness of endometrium; TM, thickness of anterior lower uterine segment myometrium*.

### Clinical Outcomes Between the Two Groups

No patients were lost to follow-up during the 3 years of evaluation, and no significant differences were observed in time to β-hCG normalization, duration of hospital stay and blood loss during treatment, and follow-up between the two groups (Table 2). The maximum blood loss in the UAE group was 350 mL, and the blood lost was collected within 1 h after operation, which was defined as a failed case. The success rates of the two groups were similar, while the complication rates were significantly different (30.19 vs. 8.82%, *P* < 0.05; Table 2). After embolization, 16 patients in the UAE group presented with complications including a low-grade fever lasting 1–3 days, leg pain, and pelvic pain, whereas only three patients in the TRR group presented with a low-grade fever (Table 2). We measured the thickness of the anterior lower uterine segment myometrium and intrauterine adhesions using ultrasound scanning 3 months after the operation. The results revealed that the anterior lower uterine segment myometria of the TRR group were thicker than those of the UAE group (1.24 ± 0.58 vs. 3.70 ± 0.63, *P* < 0.05). We observed that no patients suffered from recurrent CSP after a 3-year follow-up. Thirty patients (88.24%, 30/34) in the TRR group had full-term delivery without uterine rupture, while four patients failed.

**Table 2:** Outcomes of patients with CSP treated with UAE combined with curettage or TRR.

**Variables**	**UAE group (*n* = 53)**	**TRR group (*n* = 34)**	***P***
Success rate (%)	98.11% (52/53)	100% (34/34)	1.000
Complication rate (%)	30.19% (16/53)	8.82% (3/34)	0.019
Fever (*n*)	10	2	
Heavy vaginal bleeding (*n*)	1	0	
Pain (*n*)	9	0	
Poor healing (*n*)	0	1	
Time to β-hCG normalization (d)	30.98 ± 7.26	30.00 ± 6.12	0.159
Duration of hospital stay (d)	4.09 ± 1.27	4.26 ± 1.19	0.534
Blood loss (mL)	34.33 ± 51.69	38.02 ± 27.32	0.703
Intrauterine adhesions (*n*)	2/53	0/34	0.518
Restored menses (*n*)	52/53	34/34	1.000
TM	1.24 ± 0.58	3.70 ± 0.63	0.000

*TM, thickness of anterior lower uterine segment myometrium. Complications include fever, vomiting, heavy vaginal bleeding, hematoma, retained CSP, infections associated with vaginal incisions, poor healing, bladder trauma, vesicovaginal fistulas, and pain*.

### Cost Analyses of Patients With CSP Treated With UAE Prior to Curettage or TRR

The direct medical costs in both groups accounted for the largest proportion of the total costs. A comparison of the total costs between two groups revealed that the total cost of the UAE group was significantly higher than that of the TRR group (13,765.89 ± 2,029.12 vs. 9,063.82 ± 954.67, *P* < 0.05; Table 3).

**Table 3:** Cost analysis of patients with CSP treated with UAE before curettage or TRR.

**Variables**	**UAE group (*n* = 53)**	**TRR group (*n* = 34)**	***P***
Direct medical costs (Chinese yuan)	13,456.32 ± 2,040.91	8,776.92 ± 952.87	0.000
Indirect costs (Chinese yuan)	308.92 ± 59.59	286.97 ± 43.38	0.060
Total cost (Chinese yuan)	13,765.89 ± 2,029.12	9,063.82 ± 954.67	0.000

## Discussion

CSP has received considerable attention because it poses a life-threatening risk. CSP II may even pose a greater risk of catastrophic complications, such as uncontrolled hemorrhage and uterine rupture. CSP II refers to a deep implantation of the amniotic sac in a cesarean scar defect with progression toward the myometrium, and a thin or absent uterine myometrium between the GS and the bladder wall. UAE combined with MTX not only inhibits blood flow to the CSP but also has a direct embryo-toxic effect (9, 10). UAE combined with MTX and curettage has been widely used in the treatment of CSP due to its minimal invasion characteristic and efficiency (11, 12).

In the present study, the efficacy, safety, and the total cost of TRR of uterine defect for CSP II were evaluated and compared to those of UAE and curettage. We established that the success rate, average blood loss, duration of hospital stay, and time to serum β-hCG normalization were similar between the two groups. However, the complication rates between the two groups (30.19 vs. 8.82%, *P* < 0.05) were significantly different. In addition, 16 patients in the UAE group presented with complications, while only three patients in the TRR group presented with low-grade fever. The anterior lower uterine segment myometria of patients in the TRR group became thicker than those of patients in the UAE group after the operation (1.24 ± 0.58 vs. 3.70 ± 0.63, *P* < 0.05). The direct medical costs accounted for the largest proportion of the total costs in both groups. A comparison of total costs between the two groups revealed that the total cost of the UAE group was significantly higher than that of the TRR group (13,765.89 ± 2,029.12 vs. 9,063.82 ± 954.67, *P* < 0.05). The results revealed that 30 patients (88.24%) in the TRR group had full-term delivery without uterine rupture after a 3-year follow-up.

The efficacy of TRR of uterine defect and UAE for CSP II was compared in the present study, and the results revealed that both treatment methods achieved satisfactory success rates, although we concluded that TRR of uterine defect was more effective, with relatively lower complication rates and associated costs. TRR confers several advantages (13). First, TRR is performed through a natural orifice of the vagina and is therefore minimally invasive. Second, the surgical procedure is simple because the transvaginal approach provides a direct path of accessing the lesion. The surgical procedure is performed under direct sight; therefore, the lesion can be completely removed even when the villi are deeply implanted in the cesarean scar. Third, cesarean scar is excised and repaired simultaneously, which promotes patient recovery and reduces the incidence of recurrent CSP. Fourth, no special preoperative preparation is required, which reduces hospitalization time and cost.

Our treatment method aims to effectively excise the gestational mass and preserve future fertility among patients with hemodynamic stability. Therefore, TRR of uterine scar defect is a feasible method to treat CSP II. The method can repair wounds, in addition to terminating pregnancy, decreasing the risk of recurrent CSP, and preserving future fertility (3, 11, 14).

Nevertheless, the present study had the following limitations. To begin with, the present study evaluated only two therapeutic approaches, and all the patients exhibited hemodynamic stability. No comparisons among other emergency conditions such as a ruptured uterus or heavy vaginal bleeding were made. UAE can prevent blood flow in patients with heavy vaginal bleeding or large uterine lesions and have the desire for future pregnancy. However, the feasibility of using TRR of uterine scar defect combined with UAE in enhancing recovery of a new incision remains unknown.

In conclusion, our study suggests that TRR of uterine scar defect is a safe and effective treatment for hemodynamically stable CSP II with highly cost-effective outcomes. TRR can ensure future fertility and prevent recurrent CSP and uterine rupture. CSP with emergency conditions such as heavy vaginal bleeding or large lesions may require prophylactic treatment to prevent blood flow to the GS.

## Data Availability Statement

The data analyzed in this study is subject to the following licenses/restrictions: The data of study are not publicly available due to ethical and legal restrictions. However, upon request, data may be available from the corresponding author on reasonable request. Requests to access these datasets should be directed to Qing Wu, okwq31@163.com.

## Ethics Statement

Ethical approval for the study was provided by the Institutional Ethics Committee of Tiantai people's Hospital of Zhejiang Province, China. All study participants provided an informed written consent included the records of hospital.

## Author Contributions

QW and SC conceived and designed the study, drafted the manuscript, supervised the study, and critically revised the manuscript. QW, SC, and GQ collected the clinical data. QW, PZ, and XW were responsible for drafting the manuscript and statistical analyses. All authors contributed equally to the revision of the manuscript.

## Conflict of Interest

The authors declare that the research was conducted in the absence of any commercial or financial relationships that could be construed as a potential conflict of interest.
